# Supplementary material for: Rapid Emergence and Evolution of SARS-CoV-2 Variants in Advanced HIV Infection
Source: bioRxiv. 2024 Jan 6:2024.01.05.574420. Preprint. [Version 1] doi: 10.1101/2024.01.05.574420 (PMC10836083; doi:10.1101/2024.01.05.574420)
Supplement: Supplement 2 [file NIHPP2024.01.05.574420v1-supplement-2.pdf]

bioRxiv preprint doi: https://doi.org/10.1101/2024.01.05.574420; this version posted January 6, 2024. The copyright holder for this preprint (which was not certified by peer review) is the author/funder. This article is a US Government work. It is not subject to copyright under 17 USC 105 and is also made available for use under a CC0 license.

Supplementary Table

HIV status	Study ID	Symptom onset date	Symptom onset ~ last pos (days)	First pos ~ last pos (days)	Number of samples sequenced	Age	Sex	Initial SARS-CoV-2 RNA (rRT-PCR Ct)	CD4 count (cells/mcL)	Plasma HIV RNA (copies/mL)	Cohort
PWH	15-030	9/1/2020	79	76	12	33	Female	22.86	1	754,000	Hospitalized
	16-036	10/4/2020	79	76	17	45	Male	21.47	1	Unknown	Hospitalized
	15-029	8/27/2020	41	37	7	43	Male	20.32	6	4,290,000	Hospitalized
	20-015	9/3/2020	20	16	8	37	Female	25.85	6	Unknown	Hospitalized
	20-017	9/28/2020	37	33	10	52	Male	19.21	9	314,372	Hospitalized
	30-014	8/6/2020	69	55	17	30	Female	24.65	18	Unknown	Hospitalized
	16-033	8/21/2020	27	15	5	44	Female	20.00	25	167,000	Hospitalized
	16-032	7/12/2020	58	22	2	36	Female	25.24	33	885	Hospitalized
	S006-001	11/22/2020	38	34	8	41	Female	32.56	43	3174	Outpatient
	30-020	10/17/2020	20	10	5	41	Male	19.78	56	358,119	Hospitalized
	16-037	10/5/2020	50	33	5	35	Female	19.80	71	Unknown	Hospitalized
	S074-001	7/3/2021	8	3	2	36	Female	30.09	136	151,722	Outpatient
	20-005	7/5/2020	5	0	1	45	Male	18.87	Unknown	Unknown	Hospitalized
	24-005	7/1/2020	8	0	1	39	Male	29.41	Unknown	Unknown	Hospitalized
	14-006	8/10/2020	10	2	1	35	Female	28.40	Unknown	Unknown	Hospitalized
	16-019	6/12/2020	19	9	3	51	Female	25.98	Unknown	Unknown	Hospitalized
	24-010	8/24/2020	44	29	8	41	Female	22.40	Unknown	Unknown	Hospitalized
	27-008	9/1/2020	38	21	3	30	Female	22.74	221	Unknown	Hospitalized
	28-006	6/11/2020	8	7	2	58	Female	29.71	228	< 400	Hospitalized
	28-004	6/9/2020	10	7	3	51	Female	20.91	329	Unknown	Hospitalized
	S009-001	11/29/2020	7	3	1	38	Male	30.57	512	143	Outpatient
	J004-001	10/8/2020	10	5	1	47	Female	26.76	724	< 400	Outpatient
PWOH	J032-001	1/25/2021	5	3	1	42	Female	31.02	-	-	Outpatient
	J054-001	4/7/2021	7	5	1	47	Male	31.07	-	-	Outpatient
	S082-001	8/16/2021	7	5	1	66	Female	27.88	-	-	Outpatient
	22-010	7/22/2020	9	2	1	45	Male	28.72	-	-	Hospitalized
	30-013	8/2/2020	9	6	1	55	Female	28.18	-	-	Hospitalized
	16-024	6/13/2020	11	0	1	49	Male	29.45	-	-	Hospitalized
	J041-001	3/7/2021	14	11	4	21	Female	22.35	-	-	Outpatient
	16-016	6/9/2020	15	5	2	63	Male	25.26	-	-	Hospitalized
	15-014	6/1/2020	18	10	3	30	Female	25.51	-	-	Hospitalized
	S013-001	12/11/2020	26	23	1	62	Female	22.74	-	-	Outpatient
	S048-001	4/26/2021	28	26	3	65	Male	25.42	-	-	Outpatient
	J039-001	3/4/2021	35	31	2	47	Female	25.62	-	-	Outpatient
	J039-006	3/14/2021	35	38	4	17	Female	24.99	-	-	Outpatient
	J039-008	3/9/2021	36	34	6	0.5	Female	26.29	-	-	Outpatient
	J039-003	3/13/2021	36	38	3	24	Female	25.24	-	-	Outpatient
	S015-001	12/14/2020	37	34	2	40	Female	26.52	-	-	Outpatient
	29-004	6/7/2020	38	33	4	78	None	21.90	-	-	Hospitalized
	S039-001	3/3/2021	41	36	3	54	Male	27.88	-	-	Outpatient
	S014-001	12/15/2020	43	41	2	44	Male	26.69	-	-	Outpatient
	J060-001	4/19/2021	44	41	3	48	Male	22.25	-	-	Outpatient
	S023-001	12/17/2020	47	43	1	51	Male	31.46	-	-	Outpatient
	29-008	7/31/2020	83	78	5	49	Female	31.97	-	-	Hospitalized
	J071-004	asymptomatic	7[#]	7	2	48	Male	25.16	-	-	Outpatient
	J039-007	asymptomatic	23[#]	23	3	15	Male	27.22	-	-	Outpatient
	J039-005	asymptomatic	25[#]	25	3	17	Female	24.63	-	-	Outpatient

[#] For asymptomatic participants, date of first positive test was used as a proxy for date of symptom onset.

Supplementary Table 1. Characteristics of study participants.
